# Monitoring and evaluation of community interventions for viral hepatitis among migrants and refugees: a Delphi-based study

**DOI:** 10.7189/jogh.15.04335

**Published:** 2025-11-14

**Authors:** Domenico Pascucci, Aina Nicolàs, Abdelrahman Taha, Jeffrey V Lazarus, Matteo Di Pumpo, Vittoria Tricomi, Francesco Di Berardino, Carlo La Vecchia, José A Perez-Molina, Giuseppe Colucci, Camila A Picchio, Angelo Maria Pezzullo, Stefania Boccia

**Affiliations:** 1 Università Cattolica del Sacro Cuore, Department of Life Sciences and Public Health, Rome, Italy; 2 Fondazione Policlinico Universitario A. Gemelli IRCCS, Rome, Italy; 3 Barcelona Institute for Global Health (ISGlobal), Barcelona, Spain; 4 CUNY Graduate School of Public Health and Health Policy, New York, New York, USA; 5 University of Milan, Department of Clinical Sciences and Community Health, Milan, Italy; 6 Hospital Universitario Ramón y Cajal IRYCIS, National Referral Centre for Tropical Diseases, Infectious Diseases Department, Madrid, Spain; 7 Instituto de Salud Carlos III, CIBER de Enfermedades Infecciosas, Madrid, Spain; 8 Foundation IRCCS Ca' Granda Ospedale Maggiore Policlinico, Division of Gastroenterology and Hepatology, Milan, Italy

## Abstract

**Background:**

Migrants and refugees in Europe carry a disproportionate burden of chronic hepatitis B and C and face barriers accessing health systems. Community-based interventions can improve screening, prevention, and care, yet no framework exists to track their performance. This study aimed to generate a consensus set of indicators for monitoring and evaluating such interventions.

**Methods:**

A scoping review of PubMed from January 2005 to June 2024 identified 70 studies and 275 candidate indicators. After removing redundancies, 38 primary and 17 additional indicators were submitted to a two-round online Delphi panel. Fourteen experts from six countries (five from Spain, three from the UK, two each from Italy and from Greece, and one each from Belgium and the USA) rated each indicator on relevance, measurability, accuracy, ethics and clarity. Indicators with >67% combined ‘agree/somewhat agree’ were revised and advanced to Round 2 (R2), and were re-rated and ranked by experts.

**Results:**

Thirty-eight primary indicators and 10/17 additional indicators advanced to R2. Fifteen indicators were re-rated in R2; none were rejected. The final set comprised 50 indicators across six domains: Prevention (six), Testing (nine), Linkage to care (six), Treatment & Care (nine), Morbidity (seven) and Health System (13). Overall combined agreement averaged 95.3% (standard deviation = 7.0), with 29 indicators achieving unanimous support. Testing and Morbidity domains showed the strongest consensus. Ranking highlighted screening acceptability, infection prevalence, rapid testing results, referral success and treatment initiation as highest priorities.

**Conclusions:**

This Delphi study delivers the first consensus-driven indicator set for monitoring and evaluating community hepatitis B/C services targeting migrants and refugees. Adoption of the 50-indicator framework, and its streamlined core set, can harmonise monitoring, guide resource allocation and strengthen data-driven progress toward elimination goals.

**Registration:**

Open Science Framework

An estimated 354 million people live with chronic hepatitis B (HBV) or C (HCV) worldwide, exposing them to cirrhosis, hepatocellular carcinoma (HCC) and premature death [1]. To curb this burden, in 2016, the World Health Organization (WHO) set a 2030 elimination target: a reduction of 90% in incident cases and 65% in mortality [2]. Yet, without intensified action, 20 million additional deaths are expected between 2015–2030 [3]. Despite major advances over the past 30 years [4], over five million European Union/European Economic Area (EU/EEA) residents still live with chronic viral hepatitis conditions [5]. Globally, HBV and HCV cause 57% of cirrhosis cases and, together with liver cancer, account for 3.5% of all deaths. The highest endemicity remains in sub-Saharan Africa and Asia, where HBV prevalence reaches 5–6 % and HCV about 2% [6] Migration from these high-prevalence regions is reshaping Europe’s epidemiology [7]. More than half of migrants living in EU/EEA originate from HBV-endemic areas and nearly 80 % from HCV-endemic countries. Among individuals arriving from intermediate- or high-prevalence settings, hepatitis B surface antigen (HBsAg) and anti-HCV positivity reach 6, and 2.3 %, respectively, with local studies reporting HBV rates up to 32 % in Italy and 21 % in Spain [8,9]. Migrants now represent around 25, and 14% of chronic HBV and HCV cases, respectively, in the EU/EEA [8].

The EU4Health co-funded Multi-country Viral Hepatitis COMmunity Screening, Vaccination, and Care (VH-COMSAVAC) project was launched in response to this challenge and in alignment with ‘Europe’s Beating Cancer’ plan [10]. It operated in Greece, Italy and Spain, where migrant and refugee communities carry disproportionate HBV/HCV burdens [11–13]. Its model combines point-of-care testing, decentralised HBV vaccination and streamlined referral to specialist services for those with reactive results, aiming to avert future morbidity and mortality driven by undiagnosed infection. Community-based delivery, combining point-of-care testing, on-site vaccination, culturally adapted education and peer-supported linkage to care, can mitigate barriers such as cost, waiting times, language, legal insecurity and stigma [6,14–19]. Consequently, such programmes often achieve both high coverage and high positivity rates [16], and can contribute to micro-elimination efforts. Micro-elimination denotes achieving hepatitis elimination targets within a clearly defined sub-population or setting through tailored interventions and metrics, as a pragmatic pathway toward national and global elimination goals [20].

Continuous performance monitoring of community-based strategies is critical to identify gaps, replicate success and assess scalability. Existing indicator frameworks, such as the WHO strategic information cascade [21], and the European Centre for Disease Prevention and Control monitoring system [22], were not specifically designed for community settings. Tailored metrics are therefore needed to capture the full pathway from decentralised screening to linkage to care in non-clinical settings.

This article describes the Delphi consensus process used to define key performance indicators for monitoring and evaluation of community-based strategies for HBV/HCV screening, prevention, and management among migrants and refugees. The resulting set of indicators seeks to support data-driven evaluation, inform policy and advance Europe’s contribution to the 2030 viral hepatitis elimination goal.

## METHODS

This study used a Delphi consensus process to identify and prioritise key performance indicators for monitoring and evaluating community-based HBV and HCV screening, prevention, and management strategies. The research protocol was registered on the Open Science Framework platform [23]. The identification of indicators for the Delphi consensus included a structured multi-step process including an initial identification of indicators through a scoping literature review, and their subsequent refinement to remove duplicates and avoid redundancy (File S1–S3 in the **Online Supplementary Document**).

### Delphi consensus process

Following the indicators selection process through the scoping review, each indicator was categorised into one of the following health domains: prevention, testing, treatment and care, morbidity, and health system. These were selected among those used by WHO in its technical guidelines for monitoring and evaluating HBV and HCV [21], based on their relevance to evaluating community-based interventions. From the candidate indicators identified by the scoping review, we created:

(i) a primary list of indicators judged non-redundant and broadly feasible for community monitoring

(ii) an additional list of indicators that were distinct but potentially less feasible for routine capture, less frequently reported, or more context-specific.

Separating these lists had the rationale of reducing respondent burden. The additional list was compiled from the same scoping review (not external sources).

Experts in viral hepatitis, community-based programmes, migrant health, and public health, were recruited through a convenience sampling approach. Participation was voluntary, anonymous, and confidential, with 15-day response windows (extended by 16 and one day in the two rounds, respectively). Only complete responses were analysed.

#### Delphi method data collection

A modified Delphi approach was applied, replacing the exploratory first round with a scoping review to generate an initial list of indicators for evaluation [24].

The study followed two consultation rounds conducted using the online platform SurveyMonkey. In Round 1 (R1), panellists rated the indicators based on the following criteria: relevance, measurability, accuracy, ethical considerations, and comprehensibility (**Box 1**). They were asked to use a four-point Likert scale (‘agree’, ‘somewhat agree’, ‘somewhat disagree’, and ‘disagree’) with an additional option for ‘not qualified to respond’ [25–27]. Sociodemographic and professional characteristics of panellists were collected in R1.

Box 1 **Rating criteria for the indicators’ prioritisation exercise**

**Relevant:** In a community setting, a suitable indicator must have a robust clinical and/or empirical basis justifying its use. It should provide valuable insights relevant to various stakeholders engaged in community-based practice and policy, thereby fostering efficient actions within this context.

**Measurable:** Within a community framework, the data necessary for evaluating the indicator should be easily obtainable. This ensures that community-based programmes can regularly assess and monitor their progress.

**Accurate:** A proper indicator in a community setting should reveal significant variations in the delivery of care (sub)-processes across different community services and/or regions. These differences must be meaningful and not merely the result of random fluctuations or characteristics of the community members.

**Ethical:** In a community context, the collection, management, and analysis of indicator data must uphold the individual rights to confidentiality and informed consent, and respect the freedom of choice regarding data provision. This is particularly important in community settings where there's a close interaction with individuals and their personal data.

**Comprehensible:** An indicator in a community setting should be straightforward, ensuring that its interpretation is easily comprehensible not only to experts and stakeholders but also to the general community population. This clarity is crucial for effective communication and engagement in community-based initiatives.

Indicators that achieved <67% [26,28] agreement for responses of ‘agree’ or ‘somewhat agree’ (combined agreement) in R1 were excluded from further consideration. Those exceeding this threshold proceeded to Round 2 (R2). Open-ended feedback from experts was revised and evaluated by the research team and used for the refinement of indicators before R2. During R1, panellists evaluated the additional list of indicators, using a yes/no format to determine whether it should be included in the main list. Items with at least 67% affirmative votes were added and subsequently fully rated in R2, alongside substantially revised items from R1.

Only R1 respondents that had completed the survey were invited to R2. In R2, indicators that had undergone substantial changes following R1 feedback and those selected from the additional list were subjected to re-evaluation (re-rating) to determine their suitability for inclusion in the final list. Separately, panellists were asked to rank the indicators within each health domain based on their practical applicability by arranging them in order of relevance. Experts could choose the option ‘I prefer not to rank this indicator’ and were given the opportunity to suggest minor edits that enhance clarity. Feedback requiring substantial modifications was not considered.

#### Delphi data analysis

Each indicator was evaluated in five predefined dimensions using a 4-point Likert scale (with assigned scores from 1 (‘Disagree’) to 4 (‘Agree’) and the option to select ‘Not qualified to respond’). For each indicator, the five item scores were summed to calculate an individual total score per panellist (5–20). The total score was mapped onto one of the four predefined categories by dividing the score range (5–20) into four equal intervals of 3.75 points. When panellists selected ‘Not qualified to respond’, those responses were excluded from the calculation, and the score range was proportionally adjusted. The distribution of individual scores across the four agreement categories was calculated to assess the overall level of agreement for each indicator. This grading approach follows the convention of previous Delphi studies (34–36), with 'U' indicating unanimous agreement (100%), 'A' for 90–99%, 'B' for 78–89%, and 'C' for 67–77% [25–27].

For the ranking process, the mean score was used to summarise the scores assigned to each indicator. The ranking was structured for indicators with the lowest mean scores – indicating greater perceived importance – were assigned higher positions. If panellists selected ‘I prefer not to rank this indicator’, the lowest possible ranking value was assigned to those responses. The three indicators in each domain with the highest ranking were selected to define a core indicator set, that might serve as a proxy for wider performance in the specific domain when all indicators tracking is not feasible. No subgroup-specific analyses (*e.g*. based on professional background or discipline) or weighting of responses were applied during the data analysis. All experts’ ratings and rankings were considered equally in the calculation of agreement levels and final indicator prioritisation.

The DELPHISTAR guideline and its compiled checklist was used for reporting (File S4 in the **Online Supplementary Document**) [29]. No external consultation occurred regarding the methodology.

## RESULTS

### Scoping review

**Figure 1:**
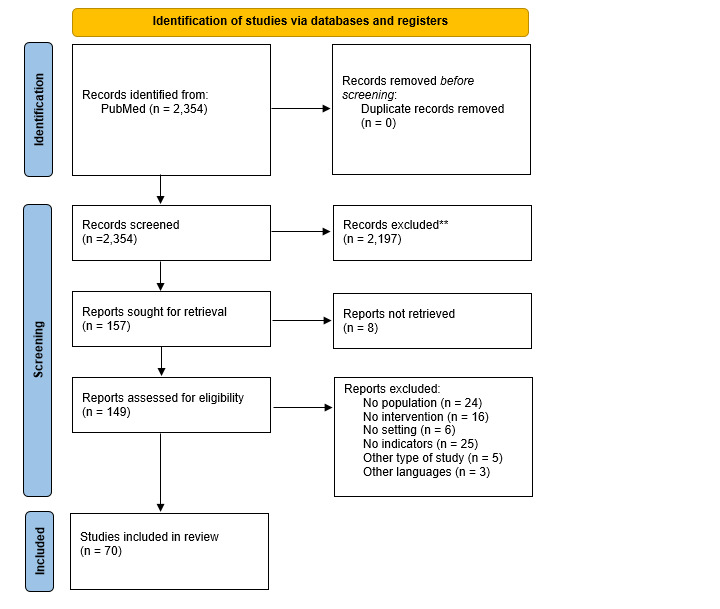
PRISMA flow diagram for the scoping review [30].

The literature search identified 2354 studies, of which 157 articles underwent full-text review. Eighty-seven articles were excluded and 70 studies were included for analysis (**Figure 1**), from which 275 indicators were extracted (File S5 in the **Online Supplementary Document**). Following their revision, a preliminary list of 38 indicators and an additional list of 17 indicators were included for expert evaluation (File S6 in the **Online Supplementary Document**).

### Results of the Delphi consensus study

Following the selection process, the preliminary set of indicators was organised into six key health domains: prevention (six), testing (six), linkage to care (eight), treatment and care (seven), morbidity (five), and health system (six). The 17 indicators of the additional list were grouped into prevention (four), testing (three), morbidity (one), linkage to care (one), treatment and care (three), and health system (five).

#### Round 1

Round 1 took place from 6 December 2024 to 6 January 2025. Forty-one experts, previously identified by the research group, were invited to participate, with a response rate of 85% (35/41 experts), and 14 experts (34%) provided complete responses and were therefore considered in the analysis. Sociodemographic and professional characteristics of panellists (n = 14) were collected (File S7 in the **Online Supplementary Document**).

All 38 indicators in the preliminary main list received 67% or more agreement in responses marked as 'agree' or 'somewhat agree' (A + SA) in R1, with an overall average agreement of 93.8% (standard deviation (SD) = 8.2%). Across domains, Morbidity achieved the highest agreement with a mean of 100.0% (SD ± 0.0%). Health System recorded the lowest with a mean of 84.5% (SD = ± 7.6%). Twenty indicators reached the highest level of agreement (100%). The indicators with the lowest proportion of agreement were ‘Cost per test-positive participant’, ‘Risk behaviour’, ‘Sustained Virologic Response at 12 weeks/24 weeks’ and ‘Sampling rate’, all with 78.6% agreement. Among the additional indicators, 10/17 indicators received at least 67% affirmative votes and were added to the main list. Overall, seven indicators were dropped after R1 (no indicator from the main list and seven from the additional list) (File S8 in the **Online Supplementary Document**).

Regarding the open-ended feedback in R1, 97.4% (37/38) received qualitative comments. Of these, eight were suggested for reallocation to a different domain and 27 were suggested minor modifications, including modifications in their names or definitions, which did not imply changes in their meaning or calculation. Following researchers’ revision, two indicators, ‘HBsAg-Positive participants with detectable HBV-DNA’ and ‘Prevalence of active HCV infection’, were reassigned from the Testing to the Morbidity domain. Further, following experts’ feedback, to clarify the role of DNA/RNA testing in community settings, two new indicators were introduced: ‘HBV DNA testing’ and ‘HCV RNA testing’, and added to the Testing domain. Within the Health System domain, ‘Cultural mediators – Translators – Peer navigators’ was split into two indicators. The first, ‘Ratio of participants per cultural mediator, translator and/or peer navigator’, quantifies the number of mediators and translators involved. The second ‘Participant use of mediation services’, measures how many participants use these services.

Overall, five indicators underwent substantial modifications and 30 underwent minor modifications (File S9 in the **Online Supplementary Document**). A total of 50 indicators progressed to R2, of which 15 required rating or re-rating. The synthesis of the results of the post-R1 analysis are reported in **Table 1**.

**Table 1:** Results of the analysis of expert comments and feedback after Round 1

Domains	Indicators confirmed at first round (no substantial change)	Indicators requiring re-rating due to substantial change	First-time rating for indicators from additional list	Total number of indicators
Prevention	5	1	3	9
Testing	4	2	3	9
Morbidity	7	0	0	7
Linkage to care	7	0	1	8
Treatment and care	7	0	1	8
Health system	5	2	2	9
Total	35	5	10	50

#### Round 2

Round 2 was conducted between 7 February 2025 and 23 February 2025, and all 14 participants from R1 completed R2. All 15 indicators subjected to re-rating surpassed the supermajority threshold of A + SA, achieving a mean proportion of agreement of 97.2% (SD = ± 3.5%) (File S10 in the **Online Supplementary Document**).

Following R2, 12 indicators which underwent re-rating obtained open-ended feedback, resulting in minor edits that enhance clarity of the indicators. A final list of 50 indicators divided by the six domains was obtained (**Table 2**).

**Table 2:** Final list of indicators and their definitions divided by domain

Indicator	Definition
**Prevention**	
Education uptake	Proportion of participants who attended educational* session/s (as part of the programme) among all participants
Screening acceptability	Proportion of participants who agreed to be screened for HBV/HCV among participants who were offered screening
Self-reported previous testing	Proportion of participants who self-reported having been previously screened for HBV and/or HCV among all screened participants
Self-reported risk behaviours	Proportion of participants who self-report having engaged in risk behaviours† among all screened participants
HBV vaccination coverage (at least one dose)	Proportion of participants who received at least one dose of the HBV vaccine (as part of the programme) among those eligible for and offered HBV vaccination
HBV vaccination coverage (complete regime)	Proportion of participants who received all required HBV vaccine doses (as part of the programme) among those eligible for and offered HBV vaccination
Acceptability of resources and education programme	Median score rating (0–5) for: (1) leaflet readability, leaflet relevance, (2) acceptability questionnaire at follow-up, (3) clarity of in-person explanation, and (4) useful/relevant information of in-person explanation
Changes in knowledge and understanding (self-reported and/or questionnaire)	Proportion of participants who reported a better understanding or increased knowledge of HBV/HCV after educational session/s among all participants
Self-reported previous HBV vaccination	Proportion of participants reporting previous HBV vaccination among all screened participants
**Testing**	
Prevalence of HBV infection	Proportion of participants with a reactive HBsAg test among all screened participants
Prevalence of current or past HCV infection (based on HCV-antibody testing)	Proportion of participants with a reactive anti-HCV test among all screened participants
Participants unaware of their HBV and/or HCV status	Proportion of participants who were unaware of their status among those with a reactive HBV and/or HCV test (*i.e.* HBsAg + and/or anti HCV+)
HBV DNA testing	Proportion of HBsAg positive participants who were offered HBV DNA testing among all HBsAg positive participants
HCV RNA testing	Proportion of anti-HCV positive participants who were offered HCV RNA testing among all anti-HCV positive participants
Participant at risk of HBV infection	Proportion of participants who tested negative for HBsAg and had no evidence of past HBV vaccination nor infection (self-reported or anti-HBs/anti-HBc negative) among all screened participants
Serological evidence of previous HBV vaccination	Proportion of participants positive for anti-HBs (and HBsAg − and anti-HBc−) among all screened participants
Evidence of past resolved HBV infection	Proportion of participants testing positive for anti-HBc (within HBsAg-negative) among all screened participants
HBV immunity prevalence	Proportion of HBsAg- participants who show evidence of immunity from a past resolved infection (anti-HBc+) or from previous vaccination (anti-HBs+) among all screened participants
**Morbidity**	
Prevalence of HBV-HCV Coinfection	Proportion of participants who are simultaneously actively infected with HCV (anti-HCV+ and detectable HCV RNA) and HBV (*i.e.* HBsAg+) among all participants
Prevalence of HBV-HDV coinfection	Proportion of participants with an active HBV infection (*i.e*. HBsAg+) and active HDV infection (anti-HDV+ and HDV RNA^+^) among all participants positive for HBsAg
Prevalence of HCV-HIV coinfection	Proportion of participants who are simultaneously actively infected with HCV (anti-HCV+ and HCV RNA) and HIV among all participants with an active HCV infection (*i.e.* detectable HCV RNA)
HBsAg-Positive participants with detectable HBV-DNA	Proportion of HBsAg + participants with detectable HBV-DNA among all participants testing positive for HBsAg
Prevalence of active HCV infection	Proportion of participants testing positive for anti-HCV and having detectable HCV-RNA among all screened participants
Prevalence of end-stage liver disease	Proportion of end-stage liver disease‡ cases detected among all participants positive for HBsAg
Prevalence of liver cancer	Proportion of liver cancer (*i.e*. hepatocellular carcinoma) cases detected among all participants positive for HBsAg
**Linkage to care**	
Onsite communication of rapid test results	Proportion of participants receiving their screening test results (*i.e.* rapid test results) onsite among all screened participants
Communication of results (laboratory-based tests)	Proportion of participants receiving laboratory-based test§ results in community setting among all screened participants
Referral of positive participants	Proportion of participants with an active HBV infection (*i.e.* HBsAg+) and/or anti-HCV+ referred to specialist care among all HBsAg + and/or anti-HCV+ participants
Linkage to care among positive participants	Proportion of participants with an active HBV infection HBV infection (*i.e.* HBsAg+) or anti-HCV+ successfully linked to specialist care (with a documented first visit in specialist care) among all HBsAg + and/or anti-HCV+ participants
Linkage to care to collaborating centres	Proportion of participants with an active HBV infection (*i.e.* HBsAg+) or anti-HCV+ successfully linked to specialist care at a clinical centre linked to the project among all HBsAg + and/or anti-HCV+ participants
HBV vaccination offer	Proportion of participants offered HBV vaccination among all participants eligible for HBV vaccination
Linkage to care for post-test counselling for past resolved HBV infection	Proportion of participants with a past resolved HBV infection receiving post-test counselling¶ among all participants with a past resolved HBV infection
Additional assessment among positive participants	Proportion of HBsAg + participants having at least one documented visit in specialist care and being assessed for ALT, HBV DNA levels, and liver cancer among all HBsAg + participants
**Treatment and care**	
HCV treatment initiation	Proportion of participants with an active HCV infection (anti-HCV+ and detectable HCV RNA) who started treatment among all participants with an active HCV infection
HCV treatment completion	Proportion of participants with an active HCV infection (anti-HCV+ and detectable HCV RNA) who completed HCV treatment among all participants with an active HCV infection who started HCV treatment
HCV treatment success	Proportion of participants with an active HCV infection (anti-HCV+ and detectable HCV RNA) who finished HCV treatment and were cured among all participants with an active HCV infection who completed HCV treatment
SVR12 – SVR24 following HCV treatment	Proportion of participants with an active HCV infection (anti-HCV+ and detectable HCV RNA) who completed HCV treatment and achieved SVR at 12 and/or 24 weeks among all participants with an active HCV infection who completed HCV treatment
HBV treatment eligibility	Proportion of HBsAg + participants who meet criteria║ for initiating therapy among all HBsAg + participants
HBV treatment initiation	Proportion of HBsAg + participants initiating HBV treatment among all HBsAg + participants who met the criteria for HBV treatment
Retention in care	Proportion of HBsAg + participants with more than one appointment in specialist services between screening and end of follow-up period among all HBsAg + participants
Retention in care (six months)	Proportion of participants who had another visit (after the initial visit) six months after initial screening among all recruited participants
**Health system**	
Sampling proportion	Proportion of recruited participants among the expected number of recruited participants
Participation acceptability	Proportion of participants recruited among all participants offered the opportunity to participate
Self-reported barriers to access care	Proportion of participants self-reporting encountering at least one barrier** in accessing care services among all recruited participants
Ratio of participants per cultural mediator, translator and/or peer navigator	Ratio of participants per cultural mediator, translators and/or peer navigators involved in an intervention
Participant use of mediation services	Proportion of participants who utilised mediation services†† provided by cultural mediators, translators and/or peer navigators among the total number of participants
Intervention cost (per person)	The total cost of the project (or stratified by type of activity) divided by the total number of recruited participants
Cost per test-positive participant	The total cost of the project (or stratified by type of activity) divided by the number of positive participants
Recruitment capacity	Number of sites visited for recruitment; Types of recruitment venues; Number visits for recruitment; Time spent in for recruitment
Screening and vaccination cost (per person)	Total cost of screening and vaccination activities divided by the total number of screened and vaccinated participants

ALT – alanine aminotransferase, anti-HBs – hepatitis B surface antibody, anti-HBc – hepatitis B core antibody, HBsAg – hepatitis B surface antigen, HBV – hepatitis B virus, HCV – hepatitis C virus, HDV – hepatitis D virus, SVR – sustained virological response at 12 and/or 24 weeks

*Education session/s focused on the prevention, early screening, diagnosis, treatment, and care of viral hepatitis infections.

†Risk behaviour refer to activities or practices which increase the likelihood of acquiring HBV and/or HCV, including: unprotected sex, sharing of needles, use of non-sterilised material for *e.g*. tattoo, traditional scarring, or in medical practices.

‡End-stage liver disease refers to chronic liver failure, which usually (but not necessarily) results from cirrhosis.

§Tests requiring processing of blood samples outside immediate community setting.

*¶*Post-test counselling refers to providing guidance to participants with resolved HBV infection, including health implications and follow-up recommendations.

*║*According guidelines corresponding to the region of implementation.

**Barrier can be chosen (but are not limited to): language, cultural, financial, legal, structural and disinformation-related barriers.

††Mediation services may include translation, cultural adaptation services *etc*.

Overall, the expert panel reached unanimous (‘U’) combined agreement (‘agree’ + ‘somewhat agree’) for 29 indicators (58%) (**Table 3**). Fifteen indicators had a combined agreement between 90–99% (‘A’), and six indicators (12%) had a combined agreement threshold below 90%. The mean level of combined agreement across all domains was 95.3% (SD ± 7.0%).

**Table 3:** Final list of indicators by domain with rating and ranking summary values

Indicator	Grade*	A† (%)	SA† (%)	SD† (%)	D† (%)	NQ† (%)		Ranking‡	
**Prevention**								
Education uptake	A	71.5	21.4	0	0	7.1	4	
Screening acceptability	U	92.9	7.1	0	0	0	1	
Self-reported previous testing	B	50	35.7	14.3	0	0	9	
Self-reported risk behaviours	B	50	28.6	14.3	7.1	0	8	
HBV vaccination coverage (at least one dose)	B	64.3	21.4	14.3	0	0	2	
HBV vaccination coverage (complete regime)	A	71.5	21.4	7.1	0	0	2	
Acceptability of resources and education programme	U	35.7	64.3	0	0	0	7	
Changes in knowledge and understanding (self-reported and/or questionnaire)	U	71.4	28.6	0	0	0	5	
Self-reported previous HBV vaccination	A	64.3	28.6	7.1	0	0	6	
**Testing**								
Prevalence of HBV infection	U	85.7	14.3	0	0	0	1	
Prevalence of current or past HCV infection (based on HCV-antibody testing)	U	85.7	14.3	0	0	0	2	
Participants unaware of their HBV and/or HCV status	U	71.4	28.6	0	0	0	7	
HBV DNA testing	U	100	0	0	0	0	4	
HCV RNA testing	U	92.9	7.1	0	0	0	3	
Participant at risk of HBV infection	A	78.6	14.3	7.1	0	0	8	
Serological evidence of previous HBV vaccination	U	100	0	0	0	0	6	
Evidence of past resolved HBV infection	U	92.9	7.1	0	0	0	8	
HBV immunity prevalence	U	100	0	0	0	0	5	
**Morbidity**								
Prevalence of HBV-HCV coinfection	U	92.9	7.1	0	0	0	4	
Prevalence of HBV-HDV coinfection	U	92.9	7.1	0	0	0	6	
Prevalence of HCV-HIV coinfection	U	92.9	7.1	0	0	0	7	
HBsAg-Positive participants with detectable HBV-DNA	A	71.5	21.4	7.1	0	0	5	
Prevalence of active HCV infection	U	100	0	0	0	0	1	
Prevalence of end-stage liver disease	U	71.4	28.6	0	0	0	3	
Prevalence of liver cancer	U	85.7	14.3	0	0	0	2	
**Linkage to care**								
Onsite communication of rapid test results	U	92.9	7.1	0	0	0	1	
Communication of results (laboratory-based tests)	U	100	0	0	0	0	4	
Referral of positive participants	U	92.9	7.1	0	0	0	2	
Linkage to care among positive participants	U	78.6	21.4	0	0	0	3	
Linkage to care to collaborating centres	A	64.3	28.6	7.1	0	0	5	
HBV vaccination offer	U	92.9	7.1	0	0	0	6	
Linkage to care for post-test counselling for past resolved HBV infection	U	71.4	28.6	0	0	0	8	
Additional assessment among positive participants	A	71.5	21.4	0	7.1	0	7	
**Treatment and care**								
HCV treatment initiation	U	92.9	7.1	0	0	0	1	
HCV treatment completion	U	92.9	7.1	0	0	0	2	
HCV treatment success	U	92.9	7.1	0	0	0	6	
SVR12 – SVR24 following HCV treatment	B	78.6	0	0	0	21.4	5	
HBV treatment eligibility	A	71.5	21.4	7.1	0	0	4	
HBV treatment initiation	A	85.8	7.1	7.1	0	0	3	
Retention in care	U	57.1	42.9	0	0	0	6	
Retention in care (six months)	A	92.9	0	7.1	0	0	8	
**Health system**								
Sampling proportion	B	42.9	35.7	21.4	0	0	2	
Participation acceptability	A	78.6	14.3	7.1	0	0	1	
Self-reported barriers to access care	A	78.6	7.1	14.3	0	0	3	
Ratio of participants per cultural mediator, translator and/or peer navigator	A	64.3	28.6	7.1	0	0	4	
Participant use of mediation services	U	78.6	21.4	0	0	0	5	
Intervention cost (per person)	A	64.3	28.6	0	0	7.1	6	
Cost per test-positive participant	C	42.9	28.6	21.4	0	7.1	7	
Recruitment capacity	U	71.4	28.6	0	0	0	7	
Screening and vaccination cost (per person)	A	92.9	0	0	0	7.1	7	

A – agree, D – disagree, HBsAg – hepatitis B surface antigen, HBV – hepatitis B virus, HCV – hepatitis C virus, HDV – hepatitis D virus, NQ – not qualified, SA – somewhat agree, SD – somewhat disagree, SVR – sustained virological response at 12 and/or 24 weeks, U – unanimous

*Grades are based on the percentage of combined agreement (‘agree’ + somewhat agree’). U = unanimous (100%) agreement, A = 90–99% agreement, B = 78–89%, C = 67–77%.

†Responses are presented as percentages of the total responses.

‡Ranking scale: 1 represents the best evaluation, with higher numbers indicating progressively worse evaluations.

The Testing and Morbidity domains received the highest proportion of indicators classified as 'U' (100%), with 88.9, and 85.7%, respectively. In contrast, Prevention and Health System had the lowest proportions of indicators assigned a 'U' grade, with 33.3, and 22.2%, respectively. Five indicators received a combined agreement corresponding to a ‘B’ grade. One indicator received a combined agreement with a ‘C’ grade, cost per test-positive participant (Health System), corresponding to a 42.9% agreement.

The ranking of the indicators reflects expert-identified priorities within each domain (**Table 2**). A subset of the 18 indicators, *i.e.* the three highest-ranked per domain, is presented as a potential core set for practical application (**Box 2**).

Box 2 **List of core indicators**

**Prevention**

Screening acceptability

HBV vaccination coverage (at least one dose)

HBV vaccination coverage (complete regime)

**Testing**

Prevalence of HBV infection

Prevalence of current or past HCV infection (based on HCV-antibody testing)

HCV RNA/HBV DNA testing

**Morbidity**

Prevalence of active HCV infection

Prevalence of end-stage liver disease

Prevalence of liver cancer

**Linkage to care**

Onsite communication of rapid test results

Referral of positive participants

Linkage to care among positive participants

**Treatment and care**

HCV treatment initiation

HCV treatment completion

HBV treatment initiation

**Health system**

Sampling proportion

Participation acceptability

Self-reported barriers to access care

HBV – hepatitis B virus, HCV – Hepatitis C virus

## DISCUSSION

This study identified and prioritised a set of 50 key indicators for monitoring and evaluating community-based screening, prevention, and management interventions for viral HBV and HCV among migrants and refugees, through a Delphi consensus process. The indicators were clustered into six domains, with a consistently high level of expert agreement.

These indicators respond to calls for ‘micro-elimination’, the strategy of achieving hepatitis elimination in clearly defined sub-populations [20]. Migrants and refugees are recognised micro-elimination target populations due to their often geographically clustered presence, accessibility through community networks, and underrepresentation in national surveillance systems [31]. The indicators proposed align with the core monitoring outcomes identified by Lazarus et al. [20] for HCV micro-elimination efforts, including prevalence of infection, people living with a diagnosis, treatment initiation, and cure. These indicators can measure short-term improvements, demonstrating progress that can build momentum for broader hepatitis elimination policies. Similarly, the 2024 Lancet Commission update identified simplified models of care, better community-based diagnostic strategies, and rigorous indicator frameworks as prerequisites for closing the persistent diagnosis and treatment gaps to eliminate viral hepatitis [1]. By aligning with these priorities, the proposed indicators can support national programmes in measuring the impact of migrant-focused services, guiding resource allocation, and enabling cross-country comparisons.

The Testing and Morbidity domains recorded the highest proportion of unanimously accepted indicators, reflecting their measurability [32,33] and concordance with international guidelines [5,21]. In Prevention, indicators related to self-reported testing and risk behaviours were rated less favourably, probably to recall and social desirability biases which undermine their reliability [34]. The Health System domain showed the lowest consensus, suggesting that several practical hurdles limit the feasibility of health system indicators. Calculating denominators such as ‘expected recruits’ and ‘offered invitations’ is challenging due to fluid population movement and difficulties tracking refusals or missed contacts, and costing might be particularly complex due to fragmented funding, including in-kind contributions, and dynamic resource allocation across activities. To address these challenges, standardising the recruitment-to-treatment cascade, adopting a clear venue taxonomy, and implementing ingredients-based micro-costing with explicit allocation rules should enhance feasibility and comparability of initiatives.

The highest-ranked indicators include screening acceptability, prevalence of viral infections and liver cancer, communication of test results, referral to specialist care and treatment initiation, which mark the critical steps of the care cascade. ‘Screening acceptability’ reflects willingness to test, awareness campaign effectiveness, and persistent barriers [35,36]. Early diagnosis and timely results communication are essential to measure disease burden and ensure rapid access to care, minimising loss to follow-up and preventing disease progression [37–40]. Similarly, referral to specialist care and treatment initiation assess the capacity to link individuals to specialised management pathways which can prevent disease progression and improve health outcomes [41]. Last, ‘Prevalence of liver cancer’ reflects missed opportunities for early diagnosis and treatment, which can avert advanced disease.

Importantly, rating and ranking results for some indicators were discrepant. For instance, **‘**HBV vaccination coverage (at least one dose/complete regimen)’ were ranked high despite moderate agreement (85.7 and 92.9%, respectively). This may reflect tension between perceived strategic importance (*e.g*. high priority for elimination and equity) and perceived measurability or data quality (*e.g*. fragmented immunisation registries, incomplete documentation of doses received across jurisdictions and increased dropout risk [38]). In short, experts judged these indicators as critical to track yet recognised practical barriers to measure them reliably in routine community workflows. Similarly, ‘Sampling proportion’ was ranked as a priority, suggesting its critical role to overall success of interventions. Yet, it received lower agreement (78.6%), potentially due to uncertain denominators and difficulties in invitation tracking.

Indicators on continuity of care and treatment outcomes (*e.g.* ‘HCV treatment success’) achieved high agreement but lower rankings, likely due to feasibility limits in community settings and their long-term nature.

Research and policy documents have summarised and evaluated the best practices [42], and international [5,21], and national [43,44] monitoring of viral hepatitis, and community interventions for high-risk groups have been assessed [45–47]. However, there is a lack of a defined and validated indicator set for monitoring community-based programmes aimed at migrants and refugees. The lack of standardised monitoring tools perpetuates a fragmented and reactive response to viral hepatitis infections among these groups. In response, this study provides a structured framework to evaluate and improve services, supporting more strategic and inclusive policy. Systematic impact measurement facilitates reporting and advocacy, equipping policymakers and stakeholders with concrete data for strategic decisions and inclusive policies. Standardised indicators enhance coordination from local to international programmes, guiding resources to more cost-effective interventions. By focusing on core key performance indicators as proxies for success in each domain, evaluators can efficiently measure impact [48].

### Strengths and limitations

The major strength of this study lies in its novelty: it is the first to propose a standardised set of indicators for monitoring and evaluating community-based screening, prevention, and management of HBV and HCV among migrants and refugees. The Delphi consensus process ensures that these indicators are grounded in scientific evidence while incorporating expert opinion. However, consensus-building presents inherent challenges, particularly due to potential variability in expert perspectives and the need to balance diverse priorities within the selection process.

One key limitation concerns the expert selection process. While convenience sampling may limit diversity, the panel was selected to reflect a range of expertise across hepatitis prevention. Furthermore, response rates are often a concern in Delphi studies, and a recent systematic review [49] noted that the median number of invited experts was only 17 participants. In this study, 41 experts were invited, and 14 completed both rounds – an acceptable rate in this context. Further, the composition of the responding panel may have influenced the prioritisation of indicators, with 65% of experts being academic experts. Most respondents were based in Europe; hence, adaptations may be necessary to ensure applicability in other settings. When the expert panel is relatively small, predominantly academic, and concentrated in a geographical region, biases in the prioritisation of indicators are likely, with preference for research-friendly, publishable indicators and underweighting of operational constraints faced by frontline implementers. However, implementation-oriented indicators were prevalent in the list and there was no sign of prioritisation of research-friendly, ‘publishable’ indicators over the latter. Field validation and possible re-weighting and adaptation by practitioners in diverse settings is desirable. Finally, the iterative Delphi process allowed experts to reassess and refine evaluations, helping mitigate biases and enhance robustness. The initial scoping review was limited to PubMed and English-language publications. This choice, made to ensure manageable screening workload, have excluded grey literature and non-English reports, thus reducing coverage of community programme reports and country guidelines published in other languages. As indicators were drawn from the literature, some metrics not reported in the literature may have been overlooked, however, to counteract, panellists were allowed to propose additional indicators or suggest substantial revisions.

### Future considerations

Future research should validate these indicators in operational programmes, with particular attention to health-system and linkage-to-care metrics, where data availability and interoperability differ widely. Regional adaptations may be necessary to reflect variations in health policy, access barriers and disease burden. Lastly, implementation studies should quantify the resources required to collect each indicator to ensure sustainability in low-resource settings.

## CONCLUSIONS

This consensus-based framework offers a practical, evidence-grounded tool for monitoring community-based HBV/HCV interventions targeting migrants and refugees. The 18-indicator core set identified by the panel can be sufficient to measure performance across the six domains where resources are sparse, while the more complete 50-indicator set can be used for more in-depth reviews or adapted following contextual needs. Further validation is needed to assess their applicability across diverse contexts, yet these indicator sets provide a structured framework for improving data-driven decision-making and optimising community-based strategies. Their adoption can enhance the effectiveness of viral hepatitis screening, prevention, and management strategies, contributing to broader elimination efforts.

## Additional material


Online Supplementary Document
