# Supplementary material for: Monitoring and evaluation of community interventions for viral hepatitis among migrants and refugees: a Delphi-based study
Source: J Glob Health. 2025 Nov 14;15:04335. doi: 10.7189/jogh.15.04335 (PMC12616579; doi:10.7189/jogh.15.04335)
Supplement: Online Supplementary Document [file jogh-15-04335-s001.pdf]

Supplement to: Pascucci D, Nicolàs A, Taha A, Lazarus JV, Di Pumpo M, Tricomi V, Di Berardino F, La Vecchia C, Perez-Molina JA, Colucci G, Picchio CA, Pezzullo AM, Boccia S. Monitoring and evaluation of community interventions for viral hepatitis among migrants and refugees: a Delphi-based study. J Glob Health. 2025;15:04335.

Domenico Pascucci[1,2*], Aina Nicolas[3*], Abdelrahman Taha[1], Jeffrey V Lazarus[3,4], Matteo Di Pumpo[1], Vittoria Tricomi[1], Francesco Di Berardino[1], Carlo La Vecchia[5], José A Perez-Molina[6,7], Giuseppe Colucci[8], Camila A Picchio[3#], Angelo Maria Pezzullo[1#], Stefania Boccia[1,2#]

1. Section of Hygiene, Department of Life Sciences and Public Health, Università Cattolica del Sacro Cuore, Rome, Italy
2. Fondazione Policlinico Universitario A. Gemelli IRCCS, Rome, Italy
3. Barcelona Institute for Global Health (ISGlobal), Barcelona, Spain
4. CUNY Graduate School of Public Health and Health Policy, New York, NY, USA
5. Department of Clinical Sciences and Community Health, University of Milan, Milan, Italy.
6. National Referral Centre for Tropical Diseases, Infectious Diseases Department, Hospital Universitario Ramón y Cajal IRYCIS, Madrid, Spain
7. CIBER de Enfermedades Infecciosas, Instituto de Salud Carlos III, Madrid, Spain
8. Division of Gastroenterology and Hepatology, Foundation IRCCS Ca' Granda Ospedale Maggiore Policlinico, Milan, Italy

* co-first authors

\# co-senior authors

Correspondence to: Angelo Maria Pezzullo, MD, MSc, PhD (angelo.pezzullo@unicatt.it)

1. **File S1: Amendment to the protocol**
2. **File S2: Methods: Scoping literature review**
3. **File S3: Systematic step-by-step list for the Delphi process**
4. **File S4: Delphi studies in social and health sciences – recommendations for an interdisciplinary standardized reporting (DELPHISTAR) checklist (link to the Word document)**
5. **File S5: List of articles and initial list of indicators (link to the Excel workbook)**
6. **File S6: List of indicators for Round 1 (link to the Excel workbook)**
7. **File S7: Characteristics of panellists**
8. **File S8: Results of Round 1 (link to the Excel workbook)**
9. **File S9: List of indicators for Round 2 (link to the Excel workbook)**
10. **File S10: Results of Round 2 (link to the Excel workbook)**

1. File S1: Amendments to the protocol – November 27, 2024

Monitoring and evaluation of viral hepatitis B and C screening, prevention and management: a protocol for prioritisation of performance indicators for community programmes targeting migrants and refugees.

Published on OSF on 5 November 2024, and accessible at https://osf.io/m7jz3/

All changes are listed below

Manuscript section	Amendment/Clarification	Rationale
Data extraction	Indicators will be identified by thoroughly reviewing all sections of each article, including the abstract, methods, results, and discussion. Specific attention will be given to any explicitly reported indicators, as well as data points that could be used to derive relevant measures. Furthermore, tables, figures, and supplementary materials will be examined to ensure a comprehensive extraction of indicators.	The amendment clarifies the indicator extraction process by specifying a comprehensive review of all article sections, including tables and supplementary materials.
Prioritization of indicators	To enhance the rigor and comparability of this Delphi study, the DELPHISTAR reporting guideline will be applied, following its recommendations for structured reporting in health and social sciences research	The amendment includes a reference to the DELPHISTAR reporting guideline to ensure standardized and transparent reporting of the Delphi process.
Delphi method data collection	Indicators that achieved 67% or less agreement for responses of "agree" or "somewhat agree" (combined agreement) in R1 will be excluded from further consideration. Conversely, those exceeding this threshold will proceed to R2. Indicators that do not undergo substantial modifications will advance directly to the ranking phase, while those that undergo significant revisions will be re-evaluated through an additional rating process before moving forward.<br><br>During R1, panellists will be required to assess whether the indicators from the additional list—representing those for which the core group has not reached a consensus—should be incorporated into the main list. This evaluation will be conducted	The amendment provides a clearer specification of the rating and ranking process, detailing how indicators progress through different evaluation phases and how panellists assess their relevance and applicability.

	using a simple yes/no response format. Indicators receiving at least 67% affirmative votes will be included in the main list and rated further in R2.<br><br>In R2, panellists will review the revised indicators along with summaries outlining the modifications made. Indicators that undergo substantial changes following R1 feedback, as well as those selected from the additional list, will be subjected to re-evaluation. Using the same four-point Likert scale as in R1, panellists will reassess these indicators to determine their suitability for inclusion in the final list. Separately, panellists will rank the indicators within each health domain based on their practical applicability in community-based programs for viral hepatitis B and C screening, prevention, and management among migrants and refugees.<br><br>For the ranking, panellists will arrange the indicators in order of relevance, from the most to the least important.. If an indicator is considered not relevant, they will have the option to choose 'I prefer not to rank this indicator.' Experts will be given the opportunity to suggest minor edits to enhance the clarity of the indicator without changing its meaning, while feedback requiring substantial modifications will not be taken into account.	
Delphi data analysis	The quantitative analysis of R1 and R2 results will focus on the rating of each indicator, based on responses provided by panelists using a 4-point Likert scale. Each panelist will rate the indicator across the five predefined items (Box.1), assigning scores from 1 ('Disagree') to 4 ('Agree'), with an additional category for 'Not qualified to respond'.<br><br>To obtain a single summary measure reflecting each participant's overall assessment of the five evaluation criteria, a total score for each expert will be calculated for each indicator. Each indicator will be rated across five items (Box 1) using a 4-point Likert scale (1 = "Disagree", 4 = "Agree"), and the scores assigned to the five	The amendment provides further details on the calculation of rating and ranking, specifying how standardized indicator-level scores are derived, how agreement levels are categorized, and how ranking is determined based on mean scores.

items will be summed to generate an individual total score (for each expert). This score will range from a minimum of 5 (all responses “Disagree”) to a maximum of 20 (all responses “Agree”).

To classify the final overall level of agreement, the total score of each expert (for each indicator) will be mapped onto one of four predefined agreement categories (“Agree”, “Somewhat agree”, “Somewhat disagree”, “Disagree”). This classification will be carried out by calculating the score range and dividing the total interval (5–20) into four equal segments of 3.75 points, each corresponding to a specific level of agreement.

A detailed example of the scoring system is provided below:

- The total score will range from 5 (all responses “Disagree”) to 20 (all responses “Agree”)
- Range = 20 – 5 = 15
- Each agreement category will correspond to an equal range of 15 / 4 = 3.75 points

Agreement categories and score thresholds:

- Agree: 16.25 ≤ Score ≤ 20.00
- Somewhat agree: 12.50 ≤ Score < 16.25
- Somewhat disagree: 8.75 ≤ Score < 12.50
- Disagree: 5.00 ≤ Score < 8.75

If a participant selects the “Not qualified to respond” option for one or more items, those responses will be excluded from the total score calculation. In such cases, the maximum possible score range will be proportionally adjusted according to the number of valid responses, to ensure consistency in the interpretation of agreement levels across indicators with different response counts.

In addition, once the individual total scores are obtained, the percentage of responses falling into the categories “Agree”, “Somewhat agree”, “Somewhat disagree”,

	"Disagree", or "Not qualified to respond" will be calculated for each indicator to assess the overall level of agreement.	

2. File S2. Methods: Scoping literature review

Search strategy

A scoping review identified indicators for monitoring and evaluating HBV and HCV screening, prevention, and management in community-based strategies among migrants and refugees; HIV programme indicators were also examined for transferable insights. Community-based interventions were defined as those providing decentralized healthcare services, such as communicable disease screening, outside formal healthcare settings. These efforts, incorporating preventive measures, aim to improve public health responses and complement facility-based care, which typically emphasizes on curative and rehabilitative services (1). The review followed the Arksey and O'Malley framework (2) and the Joanna Briggs Institute manual (3).

The population, concept, and context (PCC) framework (4) was adopted to frame the following guiding question of the scoping review: "What performance indicators were being used or had the potential to be used for monitoring community-based HBV and HCV screening, prevention, and management strategies targeting migrants and refugees?

The scoping review was carried out by querying the PubMed database for articles published between January 2005 and June 2024. The search string was constructed by combining the following keywords: "hepatitis B", "hepatitis C", "HCV", "HBV", "viral hepatitis" "indicat*", "assess*", "perform*", "impact*", "evaluat*", "monitor*", "diagnos*", "prevent*", "screen*", "control*", "treat*", "vaccine*", "test*", "care", "linkage to care", "refer*", "cascade", "promot*", "commun*", "outreach*", "reach*", "decentral*", "neighbor*", "mobil*", "migra*", "migration", "immigra*", "FGI" "FGIs", "settl*", "foreign*", "displac*", "asyl*", "seek*" and"refug*"using Boolean operators "AND" and "OR" (Table 1).

Table 1. Search string used in PubMed for the scoping review

**Search string**
("HBV" [Title/Abstract] OR "HCV" [Title/Abstract] OR "Hepatitis B" [Title/Abstract] OR "Hepatitis C" [Title/Abstract] OR "Viral hepatitis" [Title/Abstract] OR "HIV" [Title/Abstract] OR "Human Immunodeficiency virus" [Title/Abstract])
AND ("assess*" [Title/Abstract] OR "perform*" [Title/Abstract] OR "indicat*" [Title/Abstract] OR "impact*" [Title/Abstract] OR "evaluat*" [Title/Abstract] OR "monitor*" [Title/Abstract] OR "effect*" [Title/Abstract])
AND ("screen*" [Title/Abstract] OR "test*" [Title/Abstract] OR "diagnos*" [Title/Abstract] OR "detect*" [Title/Abstract] OR "prevent*" [Title/Abstract] OR "control*" [Title/Abstract] OR "vaccin*" [Title/Abstract] OR "treat*" [Title/Abstract] OR "care" [Title/Abstract] OR "linkage to care" [Title/Abstract] OR "link to care" [Title/Abstract] OR "refer*" [Title/Abstract] or "cascade" [Title/Abstract] OR "promot*" [Title/Abstract])
AND ("commun*" [Title/Abstract] OR "outreach*" [Title/Abstract] OR "reach*" [Title/Abstract] OR decentral* [Title/Abstract] OR "neighbor*" [Title/Abstract] OR "mobil*" [Title/Abstract])
AND ("migra*" [Title/Abstract] OR "migration" [Title/Abstract] OR "refug*" [Title/Abstract] OR "immigra*" [Title/Abstract] OR "FGI" [Title/Abstract] OR "FGIs"

[Title/Abstract] OR “settl*” [Title/Abstract] OR “foreign*” [Title/Abstract] OR “displac*” [Title/Abstract] OR “asyl*” [Title/Abstract] OR “seek*” [Title/Abstract])

AND (2005:2024[pdat])

Reporting complied with the Preferred Reporting Items for Systematic Reviews and Meta-Analyses extension for Scoping Reviews (PRISMA-ScR) (5).

Study selection

Two groups of researchers (DP and FDB; AN and VT) independently screened titles and abstracts; full-text evaluations were then divided among the four researchers, and disagreements were resolved by a third reviewer (CAP). Inclusion criteria required studies published in English from 2005 onwards, focusing on community-based interventions for HBV, HCV, or HIV, targeting migrants or refugees, and including at least one well-described performance indicator. Articles not meeting these criteria or with incomplete data were excluded. Rayyan software (6) was used for screening.

Data extraction

Two reviewers (DP and AN) independently extracted the data using an Excel sheet, recording bibliographic details and country, disease focus, target population, indicator title with a brief description if available. All indicators that were explicitly described in the selected studies, as well as those derived from available reported data, were extracted. Tables, figures, and supplementary materials were also examined.

Selection of initial performance indicators

Following extraction of indicators from the literature, those indicators that were semantically similar or calculated using analogous methods were eliminated to avoid redundancy. Indicators that were considered irrelevant to the community setting were also discarded. All decisions were made through consensus among two independent reviewers (DP, AN) to ensure clarity and precision in the initial set of indicators to be submitted to the Delphi process. When unsure about their inclusion, indicators were set aside to compile an additional list, and later, during the Delphi process, experts were consulted about their inclusion.

Bibliography

1. Rahajeng E. Framework on Community Based Intervention to Control NCD Risk Factors [Internet]. APEC Secretariat; 2014 [cited 2025 Mar 3]. Available from: https://www.apec.org/docs/default-source/Publications/2014/8/Framework-on-Community-Based-Intervention-to-Control-NCD-Risk-Factors/FRAMEWORKS_ForPrint_OKE_2.pdf

2. Arksey H, O’Malley L. Scoping studies: towards a methodological framework. International Journal of Social Research Methodology. 2005 Feb;8(1):19–32.

3. The Joanna Briggs Institute. The Joanna Briggs Institute Reviewers’ Manual 2015: Methodology for JBI Scoping Reviews [Internet]. 2015 [cited 2025 Feb 4]. Available from: https://reben.com.br/revista/wp-content/uploads/2020/10/Scoping.pdf

4. University of South Australia. Apply PCC [Internet]. [cited 2025 Feb 6]. Available from: https://guides.library.unisa.edu.au/ScopingReviews/ApplyPCC

5. Peters MDJ, Godfrey CM, Khalil H, McInerney P, Parker D, Soares CB. Guidance for conducting systematic scoping reviews. Int J Evid Based Healthc. 2015 Sep;13(3):141–6.

6. Rayyan [Internet]. Available from: https://rayyan.ai

3. File S3: Systematic step-by-step list for the Delphi process

To enhance clarity andreadability, the key steps undertaken during each phase of the Delphi process are systematically presented below.

Round 1 (R1)

- Panellists rated the preliminary main list of indicators using a four-point Likert scale.
- Indicators that received more than 67% combined agreement (“agree” or “somewhat agree”) proceeded to R2, while those with 67% or less were excluded from further consideration.
- Panellists were also asked to evaluate a separate set of indicators—the *additional list*—which included indicators for which the core research team had not reached consensus during the preparatory phase.
    - These additional indicators were assessed using a yes/no format to determine whether they should be included in the main list.
- Indicators from the additional list that received at least 67% affirmative votes from the panel were added to the main list and included in R2.Sociodemographic and professional characteristics of panellists (e.g., gender, age, country, job category, and level of expertise) were systematically collected during R1.Panellists also had the opportunity to provide open-ended feedback to support indicator refinement, if deemed necessary.

Between R1 and R2

- We reviewed expert feedback and revised indicators accordingly.
- Indicators with only minor edits (e.g. wording or clarity) were not re-rated but moved directly to the ranking phase.
- Indicators that underwent substantial revisions (e.g. changes in meaning or structure) were flagged for re-evaluation**,** through an additional rating process**,** in R2.

Round 2 (R2)

- Panellists reviewed summaries of the modifications made. The feedback was aggregated across all expert groups.
- Indicators that had undergone substantial changes after R1, along with those newly added from the additional list, were re-evaluated using the same four-point Likert scale.
- Separately, panellists were asked to rank the indicators within each health domain based on their practical applicability in community-based HBV/HCV programmes for migrants and refugees.
    - Panellists arranged the indicators in order of relevance, from the most to the least important.
    - If an indicator was considered not relevant, they could select the option “I prefer not to rank this indicator.”
- Experts were allowed to suggest minor wording edits to improve clarity, while feedback requiring major revisions was not considered at this stage.

4. File S7: Characteristics of panellists *(n=14)*

**Characteristic**	**n (%)**
Gender	
Female	10 (71%)
Male	4 (29)
Age	38 (45 - 30)*
Country of employment	
Spain	5 (36)
UK	3 (21)
Italy	2 (14)
Greece	2 (14)
Belgium	1 (7)
USA	1 (7)
Primary sector of employment	
Academic	9 (65)
Public	3 (21)
Civil society	2 (14)
Primary field of work	
Public Health	6 (43)
Clinical Research	5 (36)
Clinical Practice	3 (21)
Years of experience in the field	
1 - 5	4 (29)
6 - 10	3 (21)
11 - 15	4 (29)
16 - 20	0

> 20	3 (21)
** Median and interquartile range*	
